# Special Issue: “Surface Engineering and Coating Technologies for Corrosion and Tribocorrosion Resistance”

**DOI:** 10.3390/ma15082885

**Published:** 2022-04-14

**Authors:** Yong Sun

**Affiliations:** Faculty of Computing, School of Engineering and Sustainable Development, Engineering and Media, De Montfort University, Leicester LE1 9BH, UK; ysun01@dmu.ac.uk

The corrosion of a material results from its interaction with the surrounding environment, which can lead to physical and chemical changes in the material and the loss of its functional properties. In fact, corrosion is one of the most damaging and costly material degradation problems in industry. It leads to economic losses equivalent to 3–4% of the GDP of an industrialised country each year. Since corrosion is a surface-related material degradation phenomenon, the corrosion performance of a material in a specific environment depends very much on the surface condition of the material. Thus, surface engineering and coating technologies have long been used to tackle the corrosion problems of a wide range of engineering materials.

Environmental condition plays an important role in the corrosion of materials. Many materials, including surface coatings, derive their corrosion resistance from passivity, i.e., the formation of a passive film at the surface. However, in many application situations—such as in chemical processing, or in marine and biomedical equipment and devices—chemical and mechanical actions are involved simultaneously. Any damage to the passive film due to mechanical action during service, such as sliding friction and wear, can lead to accelerated corrosion; this, in turn, can lead to accelerated wear. Thus, tribocorrosion is also a common degradation phenomenon in industry. Some efforts have also been made in recent years to tackle the tribocorrosion problems of surface coating systems. One of these issues is the sustainability of the surface layer or coating under tribocorrosion conditions, whereby material loss occurs continuously. A coating system with both good wear resistance and good corrosion resistance could obviously offer better tribocorrosion resistance to the material than one without.

A surface engineering and coating system is a composite system comprising the surface layers, the interfaces, the subsurface zone, and the substrate. Through proper design and implementation of the surface coating, interface, subsurface, and substrate as a system, the corrosion and tribocorrosion resistance of engineering materials can be considerably enhanced. Significant progress has been made in this respect in recent decades. For example, the corrosion resistance of engineering steels can be improved using: conventional electroplating or electroless plating; innovative coatings such as CVD and PVD coatings, and thermal and plasma spray coatings; and surface alloying through diffusional and metallurgical surface treatments. The corrosion and tribocorrosion behaviour of a surface-engineered material depends on many factors, including the electrochemical response of the surface to the environment, the chemical and phase composition of the coating, the density and porosity of the coating, the interface between the coatings, and the adhesion between the coating and the substrate. Thus, there is still a wide scope for further research and the development of corrosion- and tribocorrosion-resistant coating systems for engineering applications.

This Special Issue addresses some of the points raised above in several original publications. A brief description of the works published so far, which I am honoured to edit as a Guest Editor, is given below, to highlight the quality of these original works of research.

The automotive industry has used coating technologies for many years to enhance the corrosion resistance of automotive bodies, which are mostly made of steel sheets. In general, the steel sheet is surface-treated by phosphating, to improve corrosion resistance and ensure coating-film adhesion before painting. However, the quality of the phosphate treatment, i.e., phosphatability, depends on the surface condition of the steel sheet. The advanced high-strength steels (AHSSs) currently used in the automotive industry for weight reduction contain a significant amount of Si as an alloying element. A Si oxide film can form on an AHSS surface, which acts as a barrier to phosphating. In order to improve the phosphatability of AHSSs, it is necessary to remove the Si oxide from the surface using a pickling treatment. In the work reported in [1], the authors studied the effectiveness of different pickling solutions in removing Si oxide from the surface of AHSS and improving its phosphatability. Three pickling solutions were investigated, including a conventional HCl pickling solution, a HCl-based solution containing 30 wt.% NH_4_HF_2_, and a HNO_3_-based solution containing 30 wt.% NH_4_HF_2_. After pickling, phosphate treatment was performed according to a common automotive process. The results showed that SiO_2_ was effectively removed upon pickling in solutions containing NH_4_HF_2_; moreover, among the two NH_4_HF_2_-containg solutions, the HNO_3_-based solution was more effective. The optimal pickling solution was the HNO_3_-based solution with a HNO_3_ concentration higher than 13%. This optimised pickling condition provided much-improved phosphatability to the AHSS. The corrosion resistance of phosphate-treated AHSS was better when using the HNO_3_-based pickling condition than when using the HCl-based pickling condition. This research highlights the importance of pre-treatment in affecting the quality and corrosion performance of the final coating product.

Tool steels and mould steels are used to make tools, moulds, and dies for manufacturing and material processing, including cutting and forming. In use, they are subjected to severe stresses and environmental conditions, and can easily be damaged due to wear and electrochemical corrosion. Surface engineering and coatings are commonly applied to these kinds of steels to improve their performance and service life. Commonly used technologies include surface alloying using thermochemical treatments such as nitriding, CVD and PVD coatings, and duplex treatments. In the work reported in [2], the authors prepared Mo coatings on H13 mould steel using an electro-spark deposition (ESD) process, and studied the mechanical and corrosion properties of the resultant coatings. Mo coatings of thicknesses ranging from 15 µm to 50 µm were prepared through ESD, using a Mo rod as the electrode and employing various deposition powers, discharge frequencies and specific deposition times. It was found that the coating thickness generally increased with increasing deposition power and deposition time, up to a certain point. Due to the melting and fusion of the materials during the deposition process, the coating surface was relatively rough, and may have contained cracks. Under optimal conditions, it was possible to produce a coating about 35 μm thick without severe cracks. The coating contained a mixture of multi-phases, mostly intermetallic compounds between Fe, Mo and Cr. The coating possessed a high hardness of nearly 1400 HV and a good wear resistance seven times higher than that of the substrate. In particular, the corrosion resistance of the coating was much better than that of the substrate due to the excellent corrosion resistance of Mo. The combination of the improvement in hardness, wear resistance and corrosion resistance would be beneficial in enhancing the working performance of H13 steel dies and moulds in practice.

As mentioned above, the corrosion behaviour of a coated material depends on many factors, including the composition of both the coating itself and the interfacial region, i.e., the bond coat. In many applications, composite coatings with multi-elements and phases are used. The electrochemical response of different elements and phases will affect the corrosion properties of composite coatings. In the work reported in [3], the authors investigated the hydrothermal corrosion behaviour of double-layer glass/ceramic composite coatings with passive fillers. The coatings were produced via the polymer-derived ceramic (PDC) synthesis route. These types of PDC glass/ceramic coatings are suitable for the protection of stainless steel from oxidation at temperatures up to 950 °C [4]. The research presented in this work [3] is comprehensive, involving: quasi-dynamic corrosion tests under hydrothermal conditions at 200 °C, for 48–192 h; mass loss/gain measurements; the analysis of corrosion solutions using inductively coupled plasma–optical emission spectrometry; Raman spectroscopy; scanning electron microscopic examination; and EDXS and XRD analysis. The presented research provides an insight into the degradation mechanisms of coatings during various stages of the hydrothermal process. It was found that the composite coating containing polycrystalline Al_2_O_3_-Y_2_O_3_-ZrO_2_ (AYZ) as an additional filler performed the best, and no significant damage was observed after the test. This indicates the potential of the PDC coating with an AYZ passive filler for application in harsh environmental conditions.
